# Are national policies on global health in fact national policies on global health governance? A comparison of policy designs from Norway and Switzerland

**DOI:** 10.1136/bmjgh-2016-000120

**Published:** 2017-04-04

**Authors:** Catherine M Jones, Carole Clavier, Louise Potvin

**Affiliations:** 1 Chaire Approches communautaires et inégalités de santé, Montréal, Québec, Canada; 2 Institut de recherche en santé publique de l'Université de Montréal, Montréal, Québec, Canada; 3 Département de Médecine sociale et préventive, École de santé publique de l'Université de Montréal, Montréal, Québec, Canada; 4 Regroupement stratégique Politiques publiques et santé des populations, Réseau de recherche en santé des populations du Québec, Montréal, Québec, Canada; 5 Département de Science Politique, de l'Université du Québec à Montréal, Montréal, Québec, Canada

## Abstract

**Background:**

Since the signing of the Oslo Ministerial Declaration in 2007, the idea that foreign policy formulation should include health considerations has gained traction on the United Nations agenda as evidenced by annual General Assembly resolutions on global health and foreign policy. The adoption of national policies on global health (NPGH) is one way that some member states integrate health and foreign policymaking. This paper explores what these policies intend to do and how countries plan to do it.

**Methods:**

Using a most similar systems design, we carried out a comparative study of two policy documents formally adopted in 2012. We conducted a directed qualitative content analysis of the Norwegian *White Paper on Global health in foreign and development policy* and the *Swiss Health Foreign Policy* using Schneider and Ingram's policy design framework. After replicating analysis methods for each document, we analysed them side by side to explore the commonalities and differences across elements of NPGH design.

**Results:**

Analyses indicate that NPGH expect to influence change outside their borders. Targeting the international level, they aim to affect policy venues, multilateral partnerships and international institutions. Instruments for supporting desired changes are primarily those of health diplomacy, proposed as a tool for negotiating interests and objectives for global health between multiple sectors, used internally in Switzerland and externally in Norway.

**Conclusion:**

Findings suggest that NPGH designs contribute to constructing the global health governance system by identifying it as a policy target, and policy instruments may elude the health sector actors unless implementation rules explicitly include them. Research should explore how future NPGH designs may construct different kinds of targets as politicised groups of actors on which national governments seek to exercise influence for global health decision-making.

**Key questions**

**What is already known about this topic?**

- The adoption of global health strategies by some governments in European countries over the past decade represents a formalised approach to health and foreign policymaking at the national level.
- Case studies on the development of the UK's and Germany's global health strategies have contributed to understanding the motivations and interests for their production in those countries.
- Little is known about the content of these documents regarding what they propose to do from a public policy perspective.

**What are the new findings?**

Based on a comparative analysis of formally adopted national policy on global health (NPGH) documents in Norway and Switzerland:

- Actors at the international level that make policy are targets for countries to influence change related to global health;
- Health diplomacy is an instrument countries use either domestically or internationally for supporting the desired change related to global health;
- The specification of rules for implementing NPGH varies between these policy documents.

**Recommendations for policy**

- Actors in the global health governance system are the target population intended to benefit from change as a result of implementing NPGH.
- When policy instruments are unfamiliar to or outside of the mandate of actors in the health sector, the implementation of NPGH may exclude structures from the health sector unless rules specifically include them.
- An empirically informed definition of NPGH is proposed for researchers and policymakers.

## Introduction

The Oslo Ministerial Declaration^1^ signed by seven ministers of foreign affairs encapsulated ideas about how expanding the scope of foreign policy to strategically include global health on the international agenda is an important step towards improving collective action and multilateral co-operation on transnational policy issues (eg, development, environment, security) related to health. Although foreign policy and health academics and practitioners continue to reflect on the relationship of these two policy sectors and the implications for practitioners engaged in global health diplomacy fields,^2–5^ there is little in the literature to advance knowledge about what countries are doing to develop and manage policy at the interface of the fields of health and foreign affairs.^6–11^ Appearing in some high-income and middle-income countries over the past decade,^10^ ^12–15^ national policies on global health (NPGH) are one way for countries to co-ordinate and integrate health and foreign policymaking. This paper explores the general question: what do NPGH propose to do and how do they plan to do it?

We define NPGH as policies that aim to organise and co-ordinate a state's action on global health across more than one sector of public administration, as part of a coherent approach to policy development and implementation between relevant ministries involved in improving health on a global scale.^16^ Within a knowledge base about countries' motivations for integrative approaches at the national level of policymaking to develop their strategies on health and foreign policy,^10–13^ ^15^ ^17^ it is unclear what such policies intend to do about global health. The transnational dimension of global health is important because the social, political and economic causes, impacts and consequences of a health problem or solution are not contained within countries' borders.^18–20^ Some researchers suggest that the foreign policy sector plays a significant (even dominant) role in agenda setting, establishing priority interests and funding of country's work at the interface of health and foreign policy.^21^ ^22^ But little is known about which sector's expertise and what kind of policy tools are used for NPGH which seem to develop at the junction of at least three policy sectors (health, development and foreign policy) and two policy levels (domestic and international). For example, research in Canada shows that barriers to integrating health into foreign policy decision-making processes include health actors' lack of diplomatic expertise (eg, knowledge of international law, negotiation skills) and diplomatic actors' lack of health expertise (eg, knowledge of health impacts of other policies, health systems).^23^ ^24^

By questioning what NPGH intends to change and how it plans to accomplish this, we aim to better understand the multilevel and multisectoral empirical characteristics of such policies. First, NPGH requires domestic actors to collaborate to improve health globally, but it remains unspecified where change is expected (internally at the national level or externally at the international level). Second, NPGH demands that the health sector collaborates with the foreign affairs and the development sectors, but we do not know whether the goals and methods of intervention will be those of the health sector or of another sector. To this end, we study NPGH with tools of health political science, a field of research that uses theories from political science in health policy research to generate knowledge about policy change in matters related to public health.^25^

In policy science, policy design is generally conceptualised either procedurally (design process, policy formulation/experimentation, crafting policy) or substantively (outcome of design process, policy content, instrumentation).^26^ ^27^ Schneider and Ingram conceptualise policy design as the elements comprising the content of public policy.^28^ ^29^ Content includes the plans, principles and underlying discourses for a policy in their instrumental and symbolic forms, which reflects in part the politics and contexts that produced it. They propose six elements of policy design: goals, targets, instruments, implementation structures, implementation rules, and rationales and assumptions. Goals and targets are about what the policy wants to do. Goals are what will be achieved; they refer to the intentional, explicit change expected as a result of the policy. Targets are for whom the change will impact; they refer to the groups for whom the policy intends to stimulate change in capacity or behaviour. Instruments are about how it will be done. Policy instruments refer to the tools and methods to support the intended changes of the goals and targets. Implementation structures and rules are about who will do it. Structures refer to the agencies responsible for policy delivery and implementing action. Rules refer to the procedures and criteria for implementation structures to work with policy instruments. Rationales and assumptions refer to the reasons for the policy. Rationales legitimise the substance of the other elements of policy design, and assumptions support the linkages between them. Rationales and assumptions justify policy design as a whole: the course of action proposed, the tools for doing it and the relevancy of the actors responsible for delivering it. This framework allows us to analyse the content of NPGH according to a set of attributes^30^ rather than according to sector-affiliated labels, also known as adjectival policy^31^ (ie, health policy, foreign policy, development policy) or policy titles (ie, health as foreign policy).^29^ Specifically, we ask whether the texts of NPGH documents adopted in different jurisdictions present any similarities in core constitutive elements that may exemplify the logic of NPGH designs.

## Methods

We conducted a comparative study of the content in cases of NPGH policy design from two countries. In this study, we define a case of NPGH policy design as a formally adopted policy document at the highest level of government.

### Case selection and construction of comparability

Using three criteria (synchronicity of NPGH policy adoption, acknowledged contributions as a state actor in global health and analogous engagement in multilateralism), we selected cases of NPGH policy design adopted in 2012 by Norway and Switzerland from a group of four countries in Europe who have adopted such policies.^32–35^ We use a most similar systems design because these criteria relate to a comparable macro-level context for global health policy at the country level.^36^ The contemporaneous adoption of NPGH in Norway and Switzerland establishes a shared timeframe for comparison. Norway and Switzerland are also similar because they are recognised for capacity to influence global health matters given their histories of contributions to health through development co-operation.^37–40^ Both countries are active member states of international normative institutions for health and development (ie, WHO (Geneva and European Regional Office) and Organisation for Economic Co-operation and Development (OECD) Development Assistance Committee). For example, in 2012, representatives from Norwegian and Swiss governments served on the WHO Executive Board. Neither country is part of the group of major economies in whose meetings global health issues are increasingly discussed (ie, G20, G7, G8)^41^ nor of the European Union (EU), which has become an active global health actor since 2010.^42^ However, both have a close relationship with the EU (Norway via the European Economic Area, Switzerland through bilateral arrangements), especially on public health issues (eg, European Centre for Disease Control (ECDC), EU Health Programme). Although considered small developed states based on population criteria (ie, under 10 million), these two middle powers^43^ have comparable ambitions for global health and its governance within a challenging climate for governments to find balance and create synergies between bilateral and multilateral development aid in their search to increase their international status and influence on global health.^44^ Informed by Sartori's^45^ questions, we compared the NPGH policy designs from these countries to examine what these adopted policy documents convey about the intentions of these two high-income countries to work intersectorally on global health, to explore similarities or differences in their design characteristics, and to improve understanding of NPGH components towards an empirical definition of this emergent policy object.

### Materials

In line with other studies on health and foreign policies that identify a single policy document as a country's policy framework for its national global health strategy,^10^ ^15^ in 2014, we downloaded publically available English language versions of adopted NPGH policy documents from government websites of Norway^33^ and Switzerland.^34^ Noting the characteristics of NPGH documents as policies that discuss global health, are developed nationally across more than one policy sector, and adopted at the highest levels of government, we excluded intersectoral policy documents discussing global health that are not formally adopted at the highest levels, government policy documents addressing specific disease issues (eg, HIV/AIDS) and global health policy documents produced by a single policy sector (eg, global health policy of national development agency). We excluded images and illustrations from our analysis.

### Data analysis

We manually coded text of the two policy documents using Schneider and Ingram's six elements of policy design^28^ to conduct a directed qualitative content analysis.^46^ We analysed each NPGH document's design architecture individually, replicating the framework's application to each text to interpret the empirical expressions of the theoretical design elements. We submitted each case's analysis for discussion with experts in two independent Context Advisory Groups established, respectively, for each case in 2014 as a strategy to reduce bias. Each group included CMJ, and CC and one experienced global health policy/governance researcher knowledgeable about their country's NPGH context. These consultations aimed to identify significant omissions from our understanding of their country's policy design, and they were independent from the comparative analysis conducted for this paper. Taking the separate analysis of each individual document, we subsequently analysed them side by side to explore the commonalities and differences across elements of NPGH design.

## Results

Norway's *White Paper on Global health in foreign and development policy*^33^ (approved by the Norwegian Parliament on 29 May 2012) is a 47-page document organised around three priority areas until 2020: mobilising for women's and children's rights and health, reducing the burden of disease with emphasis on prevention, and promoting human security through health. Each area is divided into subpriorities, listing a total of 70 commitments of the Norwegian government. The *Swiss Health Foreign Policy*^34^ (approved by the Swiss Confederation's Federal Council on 19 March 2012) is a 42-page document that presents 20 objectives for a 6-year period under three areas of interest: governance, interactions with other policy areas and health issues. Both documents convey the intention to strengthen connections between different policy sectors in their country for improving consistency in the government's global health work, but the designs differ in the problematisation—which is political in the Norwegian design and administrative in the Swiss design. An overview of the comparison shows that their contents contain common types of targets, but there is variation across the other five elements of design (see Table 1).

**Table 1:** Comparing policy design elements in Norwegian and Swiss NPGH documents (Source: Authors)

	Norway^33^	Switzerland^34^
*Targets*	Global health governance system: policy venues, institutions, networks and partnerships for collective action on global health	
*Goals*	Integrate policy levels: incorporate international and Norwegian domestic objectives related to global health	Orchestrate policy sectors: institutionalise collaborative working processes for global health between multiple sectors in Switzerland
*Rationales and assumptions*	Norway can impact global health governance building on history of political leadership contributing to global health improvement	Administrative innovation can strengthen Swiss interactions in the global health governance system
Instruments	(*External*) Global health diplomacy—international co-operation/relations	(*Internal*) Global health diplomacy—interministerial co-ordination/dialogue
*Implementation structures*	Ministry of Foreign Affairs, Norwegian Agency for Development Cooperation	Federal Department of Foreign Affairs, Federal Office of Public Health, Swiss Agency for Development and Cooperation
*Implementation rules*	Political	Administrative

### Targets

The Norwegian and Swiss policies aim to act on the global health governance system by influencing change in policy venues, international institutions, networks and partnership structures where decisions about global health policy and programmes are made. The main targets for the Norwegian policy are international policymaking arenas where political and economic support are mobilised for global health such as United Nations agencies (eg, WHO, UNICEF, United Nations Population Fund (UNFPA)), the World Bank, Global Health Initiatives (GHI) (eg, Global Alliance for Vaccines and Immunizations (GAVI Alliance), Global Fund to fight AIDS, Tuberculosis and Malaria (GFATM)), financing mechanisms (eg, UNITAID, Health Results Innovations Trust Fund (HRITF), Global Environment Facility (GEF)) and multilateral partnerships (eg, Every Woman Every Child, Global Campaign for Health Millennium Development Goals (MDGs)) (see ref. 33 sections 3.2, 5.2 and pp. 18, 20–23, 25–29, 31–37, 44). The principle targets for the Swiss policy are international institutions and governance bodies such as the OECD, the Council of Europe, WHO and EU agencies (see ref. 34 pp. 18, 19, 21, 22, 24, 27, 29). These targets are systemic and international. Both NPGH aim to impact institutions and groups of actors who make decisions about collective action on global health in which their governments have vested interests and participation.

### Goals

The foremost policy goal in the Norwegian policy is to improve the continuity of domestic and international policy objectives on global health, including a synthesis of health objectives into foreign and development policy (see ref. 33 pp. 5, 7, 15, 16, 36–38). The document's account of a composite history of Norwegian leadership and political, technical and financial contributions to global health is presented to solidify global health policy as an important domestic issue (see ref. 33 pp. 7–9 and section 3). One of the intended results is to integrate Norway's commitments to the health-related MDGs (MDGs 4, 5 and 6) into their foreign policy and technical health framework (see ref. 33 pp. 9, 10, 15–23). The goal is to integrate the political and technical aspects for understanding and developing Norway's global health work at the national level among actors involved from different policy sectors and parliament, and with its partners at the global level (see ref. 33 pp. 8, 37, 46, 47).

The principle goal of the Swiss policy is to systematise an intersectoral approach to global health work across sectors within the Swiss Federal Administration. The intended result is the normalisation of interdepartmental collaboration across agencies responsible for public health, foreign affairs, development and intellectual property to make decisions about Swiss positions on matters of global health, propose instruments for institutionalised dialogue about global health across government ministries, and to standardise the procedures for intersectoral collaboration and joint decision-making on global health issues, positions and policies (see ref. 34 pp. 7, 14, 15, 33–35).

### Rationales and assumptions

Norway's history of leadership and contributions to global health as an important state actor are the cornerstone of the rationale for its policy (see ref. 33 section 2). Examples of Norway's development aid, its global health projects and its role in initiatives like GAVI and the GFATM (see ref. 33 pp. 7, 12, 20–23, 34, 44) or the Foreign Policy and Global Health Initiative (see ref. 33 pp. 11, 36, 38, 43) serve to justify the logic for continuity of Norway's political leadership in global health and the path dependence of its programmes in this domain. The Norwegian policy is premised on the assumption that there is a need to combine national responsibility with global collective action for improving health on a global scale (see ref. 33 pp. 5–7, 13, 24, 25, 43). An individual's right to health is a responsibility of national governments and health systems to ensure, but collective action at the international level is needed to support countries with limited capacity.

The underlying assumption of the Swiss policy is that working responsibly with governance of the evolving global health architecture necessitates administrative devices for structured intersectoral collaboration at the federal level (see ref. 47 p. 18, 19). The Swiss rationale is that the institutionalisation of dialogue to improve opportunities for coherence across policy sectors in the Swiss context will increase the credibility of Swiss negotiations on global health policy positions in international settings (see ref. 47 p. 9, 10, 13–15). The arguments rely on the experience of a previous agreement between the Federal Department of Foreign Affairs and the Federal Department of Home Affairs on Swiss health foreign policy objectives (see ref. 47 p. 7, 3–35] to demonstrate the success of tools for interdepartmental co-operation on matters of health and foreign policy.

### Instruments

The Norwegian policy proposes instruments of international co-operation like official development assistance, multilateral arrangements, partnerships, political networks and global health diplomacy to achieve desired changes (see ref. 33 sections 2, 5.2). Norway strategically uses diplomatic techniques to stimulate and support change in international policy settings and to demonstrate leadership and capacity for global health stewardship at the highest levels. Examples include the Oslo Declaration of Foreign Ministers in 2007, the prime minister's establishment of the Global Campaign for the Health MDGs and the Network of Global Leaders in 2007, negotiation process for the pandemic influenza framework, health diplomacy for ratification and implementation of the Framework Convention on Tobacco Control, promoting norms of rights-based approaches to health (gender equality, sexual and reproductive rights), supporting capacity building internationally for implementation of WHO global strategies, frameworks and codes of practice, or via membership on Boards of UNAIDS or UNITAID (see ref. 33 pp. 5, 7, 9, 11, 21–22, 28, 29, 32, 36, 41).

The Swiss instruments for achieving desired changes are co-ordination tools for improving interdepartmental co-operation to strengthen coherence. Communication-based instruments (ie, e-platform CH@WORLD) support information sharing across the entire Swiss public administration and the coproduction of policy guidance (see ref. 34 pp. 33, 34). These processes are carried out in bodies such as the high-level Interdepartmental Conference on Health Foreign Policy (for oversight), an executive support group (for strategic decisions), and two interdepartmental working groups on health foreign policy and on public health, innovation and intellectual property (for operational issues) (see ref. 34 pp. 34–35). A co-ordination office, staff secondments from Federal Department of Foreign Affairs to the Office of Public Health and global health policy training for diplomatic service personnel are examples of instruments to institutionalise practices that strengthen links between health and foreign policy sectors and build capacity for fostering a deeper understanding between these sectors on the ground (see ref. 34 pp. 33, 35).

### Implementation structures

The foreign affairs ministry and development agency in each country have responsibilities for implementation; however, clarity about the role of health sector agencies in implementation differs. In the Norwegian policy, the Ministry of Foreign Affairs is the main implementation structure (see ref. 33 pp. 8, 25, 43, 46, 47). Although the Ministry of Foreign Affairs and the Ministry of Health and Care Services are joint signatories of the document, the implementation roles within and between those ministries are indeterminate, and the relationships of departments, sections and subordinate agencies in those ministries and between them regarding implementation duties for Norway's NPGH are unclear (see ref. 33 pp. 8, 46).

The main implementation structures for the Swiss policy include the Sectoral Foreign Policies Division of the Federal Department of Foreign Affairs, the Federal Office of Public Health from the Federal Department of Home Affairs, as well as the Swiss Agency for Development and Cooperation (see ref. 34 pp. 7, 34, 37). The Sectoral Foreign Policies Division (Transport, Energy and Health Section) is the primary co-ordination office for the Swiss NPGH, working closely with the Federal Office of Public Health and the Swiss Development Agency (see ref. 34 p. 33).

### Implementation rules

Both documents state that no additional resources are specifically allocated for implementation; the policy is carried out with the currently budgeted resources available. The Norwegian policy's rules are unspecific. They authorise foreign policy and development actors in the Ministry of Foreign Affairs to implement existing international commitments for global health based on an approved vision up to 2020. In that time frame (extending 5 years into Sustainable Development Goals agenda), the document's section 6 ‘Perspectives on the future’ explores the challenge of further developing ‘a coherent Norwegian global health policy’ (see ref. 33 p. 46) that encompasses use of policy instruments, including those from other sectors, for a range of problems with health consequences (eg, urbanisation, climate change). Ambiguous rules for implementation in the Norwegian design render decisions about implementation procedures for using selected instruments for specific targets to the discretion of senior government officials and politicians.

The rules for implementation in the Swiss policy are administrative because they provide the procedures for working across federal departments (ie, ministries). The rules define how the co-ordination of the interdepartmental structures is part of a collaborative process for overseeing implementation of policy-related activities (eg, rules for good governance, see ref. 34 p. 13). Two interdepartmental working groups submit an annual implementation report to the Interdepartmental Conference on Health Foreign Policy, the highest decision-making body for Swiss NPGH (under the Federal Council) (see ref. 34 p. 34). While the implementation of specific initiatives under the policy may be the responsibility of individual agencies, the implementation rules require that information and progress are shared regularly with the other federal agencies and relevant stakeholders. Administrative rules for extensive consultation and large majority consensus are also deeply engrained in the overall Swiss policymaking context.

## Discussion

Exploring the multilevel and multisectoral characteristics of NPGH, we used a policy design framework to assess whether NPGH documents adopted by Norway and Switzerland shared similar design elements. The results of this comparative analysis indicate that NPGH expect to influence and affect change outside of their borders, by targeting the international level. Methods and instruments to support desired change are primarily those of health diplomacy, as tools for negotiating interests and objectives of multiple sectors on global health, for use and adaptation internally in the case of Switzerland and externally in the case of Norway.

These results have two implications for public health. First, the global health governance system encompasses the population of actors intended to benefit from changes as a result of NPGH because the Norwegian and Swiss governments aim to influence the capacity and behaviour of this system with NPGH. These are not target populations or beneficiaries of health and social policy as generally understood by people working in public health. Policy targets in both designs include groups of actors who comprise the system of global health governance, ranging from the traditional normative institution of WHO to contemporary actors like the multipartner GHI evolving since 2000 (eg, GAVI, GFATM). A plurality of actors representing competing normative frameworks vie for policy attention and resources to support respective agendas for global health action in the global health governance system.^48^ The proliferation of actors who operate in the system leads to overlapping roles and functions that create accountability issues.^49^ Leadership and authority for health at the global scale that were conventionally a responsibility of international organisations specialising in health (ie, WHO) have been challenged by the ascent of non-state actors and GHI in the global health governance system.^50^ ^51^ Although private philanthropy has historically played a role in international health agenda setting, scholars have expressed concerns about the influence of foundations and private industry actors in the global health governance system in the 21st century, including their relationships with governments, public–private partnerships and international organisations.^52–54^

Both NPGH designs portray WHO, UN agencies, the World Bank, health-related multilateral organisations, and GHI and public–private partnerships (eg, organisations in the Health 8) as key institutions for health and arenas for political mobilisation to support technical advancements in global health. Schneider and Ingram propose that groups targeted by public policy are depicted in normative terms because policy design contributes to the social construction of policy targets.^55^ The social construction of a policy target refers to whether a group is depicted as deserving or undeserving, and policy design links the behaviour or capacity of targets to the achievement of the policy's goals.^56^ The NPGH policy designs we analysed reinforce the normative underpinnings of global health governance as a system of indispensable actors operating at the international scale, and endorse the system of global health governance as a group of actors meriting state's attention. The NPGH designs construct targets that governments cannot neglect in international negotiations for health, which supports the theoretical proposition that policy designs convey messages to ‘target groups about how government behaves and how they are likely to be treated by government’.^57^ By establishing this systemic target for NPGH in their designs, countries participate in the construction of a transnational population of legitimate and powerful global health actors as the beneficiaries of NPGH, potentially also contributing to constructing other states or international civil society organisations as contenders or dependents with less political power to act in that system. Furthermore, these countries are also insiders in the institutions of that system, and the designs confer advantage to those decision-making bodies where the country is a member and intends to influence decisions on global health.

Second, the implementation of NPGH may be elusive to health sector actors. Although both NPGH designs construct similar target populations, different instruments to reach them relate to who is responsible for implementation and according to what rules. Policy instruments are technical and social devices representing knowledge about how to coerce or enable a change in the target population.^58^ They have symbolic importance for communicating the nature of the relationship between the targets and the implementers. In Norway's design, the Ministry of Foreign Affairs is the main implementation structure with authority, legitimacy and expertise to use political mobilisation and health diplomacy as instruments to affect desired change in the international system. In Switzerland's design, the instruments are structures for interministerial collaboration, with multiple implementation agencies cutting across sectoral divides because the rules about consultation and collaborative approaches impose shared responsibility at the federal level for NPGH. In neither case are the instruments specific to the health sector or to public health. Global health diplomacy is an instrument used at international and national levels.^59^ This means that health sector actors may be challenged to work with unfamiliar policy instruments unrelated to their area of expertise (eg, global health diplomacy) or be excluded from the implementation structures even if they were involved in developing NPGH with other sectors. Public health actors seeking to influence health and foreign policy are encouraged to discern ways health is framed in global health policy.^60^ A combination of a lack of familiarity, understanding or experience with diverse policy frames and instruments may limit public health actors' participation in implementation unless implementation rules in designs support a clearly defined role for them.

### Limitations

These results and their implications for public health must be considered within the methodological and theoretical limitations of our analysis. The narrow concept of policy design we used confines the scope of policy content to texts of adopted NPGH strategy documents. This is an incomplete picture of content as theorised by Schneider and Ingram. Symbolic forms of content could be collected through interviews and other materials, but our analysis focused on single policy documents from two countries. Our deductive approach to using Schneider and Ingram's policy design elements also imposed limits. By applying generic categories of design elements to explore the content of NPGH documents, we might have omitted issues of focus in global health policy. For example, our analysis excludes discussion pertaining to the meaning of global health in NPGH and global health priority topics promoted by states for attention in the system of global health governance (eg, health systems, vaccination, universal health coverage, access to medicines, maternal and child health, global health security). Research has explained why certain policy issues receive political attention over others in the global health agenda,^61^ ^62^ but such questions were not part of the framework we adopted to explore the architecture of these NPGH documents. Finally, the framework did not equip us to acknowledge the composition of policy sectors. For example, *health* and *foreign affairs* sectors are not homogenous groups of actors or expertise; diverse subgroups compose each sector (eg, foreign policy sector includes humanitarian affairs, economic development, human security, and health policy sector includes hospitals and healthcare, insurance, drugs, public health). One caveat of conducting directed qualitative content analysis of policy documents is limitation for understanding the policy processes, negotiations between sectors and subsectors, and trade-offs in the content's production.^63^ Further research on development and implementation of NPGH is needed to contextualise their elaboration and use. Aware of these limitations, we suggest this policy design framework is a theoretical tool for public health researchers to do comparative analyses of content in policy documents across jurisdictions at any level.

## Conclusion

Global health policy and governance research generally focuses on how different frames of global health construct policy problems and legitimise the knowledge, actors and resources associated with their solutions.^64–66^ Our comparative analysis of Norwegian and Swiss policy documents to better understand what NPGH propose to do suggests that these policies contribute to the construction of global health governance through its constitution as a system of policy targets. Formally adopted NPGH in Norway and Switzerland designate the actors in this system that the country considers as legitimate and authoritative groups for making decisions that impact health on a global scale and with which state actors must interact to influence these decisions. Based on these findings, we modify our definition of NPGH to: a policy that connects a country's work on global health across more than one government policy sector, in which the health sector may not have a leading implementation role, with the aim to act in and on the global health governance system.

While there is no consensus on the understanding and use of the term global health governance in scholarship or practice,^67–69^ our findings support a conceptualisation of global health governance that is multisectoral, taking place in multiple sites and on multiple levels.^66^ ^70^ This leads us to question the empirical salience of conceptual distinctions between global health governance and global governance for health^69^ as systems targeted by NPGH because the two designs target alike actors for whom health is the main objective and actors for whom it is not. NPGH targets various types of actors making decisions related to global health whether they are specialised in health or not (eg, intergovernmental, private/public, state/non-state and hybrid: see ref. 71 72 for thorough presentation). For example, the amalgamation of public and private actors in the global health governance system may be of concern for NPGH designs when policy targets that include financing mechanisms (ie, GAVI, GFATM) are combined with those that do not, or when governments target transnational public institutions similarly to private or hybrid actors. In this study, we found that NPGH designs designated different actors in the global heath governance system as targets for government attention without transparent analysis of institutional arrangements or explicit questioning of normative or evaluative bases for targeting them. Noting pressing questions regarding legitimacy and accountability of philanthropic and hybrid actors operating in the global health governance system,^73–76^ we think future research needs to explore how NPGH designs may construct different kinds of targets as politicised groups of actors on which national governments seek to exercise influence.
